# Physical-Based Simulation of the GaN-Based Grooved-Anode Planar Gunn Diode

**DOI:** 10.3390/mi11010097

**Published:** 2020-01-16

**Authors:** Ying Wang, Liu-An Li, Jin-Ping Ao, Yue Hao

**Affiliations:** 1 School of Electronic Information, Northwestern Polytechnical University, Xi’an 710072, China; yingwang@nwpu.edu.cn; 2 The State Key Discipline Laboratory of Wide Band Gap Semiconductor Technology, School of Microelectronics, Xidian University, Xi’an 710071, China; yhao@xidian.edu.cn; 3 School of Electronics and Information Technology, Sun Yat-Sen University, Guangzhou 510275, China; liliuan@mail.sysu.edu.cn

**Keywords:** wide band gap semiconductors, numerical simulation, terahertz Gunn diode, grooved-anode diode

## Abstract

In this paper, a novel gallium nitride (GaN)-based heterostructure Gunn diode is proposed for the first time to enhance the output characteristics of Gunn oscillation waveforms. A well-designed grooved anode contact is adopted to separate the long-channel diode into two short-channel diodes in parallel. If the grooved anode contact is positioned in the middle of the device, the output power nearly doubles in the grooved-anode diode compared with the single-channel ones, as does the output frequency. Based on the numerical results, the best output characteristics are obtained at the 2.0-µm symmetrical grooved-anode diode, which produces nearly 5.48 mW of power at the fundamental frequency of 172.81 GHz, with 3.13% efficiency of power conversion. If the grooved anode contact is not positioned in the middle of the diode, the harmonic frequency would be enhanced. The GaN heterostructure grooved-anode Gunn diode has been demonstrated to be an excellent solid-state source of terahertz oscillator.

## 1. Introduction

Terahertz (THz) waves (300 GHz–10 THz) have been extensively studied in recent years due to their potential applications in the fields of communication, imaging, radar, spectroscopy and security screening [1,2,3]. Terahertz has been a “research gap” for a long time. Indeed, no powerful radiation sources have been available until the last few years. From a practical point of view, solid-state devices show excellent potential as terahertz sources, which can be integrated with other electronic or optoelectronic devices within a single chip [4]. GaN-based Gunn diodes are one of the most excellent solid-state terahertz oscillators [4] and attract much interest thanks to the unique properties of gallium nitride, such as its wide band gap (3.42 eV) [5], high electron mobility [6], high breakdown field (3.3 MV/cm) [5], high thermal conductivity [7] and so on. In recent years, the research has been mainly concentrated on heterostructural planar Gunn diodes rather than the traditional vertical ones [8,9,10,11,12]. Compared with the traditional vertical Gunn diode, the heterostructural planar Gunn diode has great advantages. First of all, it allows for the easy integration with other devices in terahertz monolithic integrated circuits, as all the contacts of planar diodes are fabricated on one plane [10]. Secondly, its oscillation frequency is controllable by determining the contacts’ distance. Lastly, due to the excellent electron transport properties of the two-dimensional electron gas (2DEG), the planar Gunn diode generates a higher oscillation frequency than bulk ones [9,10,11,12]. However, on one hand, the radio frequency (RF) output power of the planar Gunn diode has been predicted to be much lower than the vertical ones [13,14,15]. On the other hand, the fundamental oscillation of Gunn diodes with a channel length of 1–2 µm reported so far have been far from the terahertz regime [8,9,10,11]. Achieving a Gunn diode with a terahertz oscillation and higher output characteristics is a worldwide problem that should be solved with great urgency. High RF power and high operation frequency seem to be two contradictory pursuits which are difficult to satisfy simultaneously. The shorter the transit region length, the higher the oscillation frequency is [16]. However, the operation bias for short-transit-region diodes is relatively low, which limits their RF output characteristics. In addition, the small-size devices also face a complicated process. Some reported planar Gunn diodes, like nanowire slot diodes and self-switching diodes (SSD) produce very high frequency oscillation, even as high as several THz [17,18,19,20,21]. Nevertheless, all of these emerging devices face the same serious problem–that is, how to generate sufficient RF power. Some theoretical work also shows other solutions, like the harmonic Gunn diode reported in [22,23] and the multi-channel Gunn diode in [24], which either suffers from low RF output power or a complicated process. In order to achieve higher frequency and higher output power simultaneously, for the first time, we propose this new-type grooved-anode Gunn diode, which is realized by simply etching a rectangle groove onto the semiconductor layer at one lithographic step. The anode contact is deposited in the rectangle groove and two cathodes are defined as being adjacent to terminals of AlGaN/GaN heterostructural channel. Therefore, one diode actually turns into two diodes placed in parallel. In the symmetric grooved-anode Gunn diode, where the length of the left and right channel is equal, the output power and oscillation frequency approximately doubles in the grooved-anode diodes compared with the single-channel ones. In the asymmetric grooved-anode Gunn diode, the harmonic frequency is greatly enhanced. In this paper, we present a detailed study into the GaN-based heterostructural grooved-anode Gunn diode based on the Silvaco simulator. We have demonstrated that it effectively improves the RF output power and operation frequency of the Gunn diode simultaneously as compared with many other structures.

The structure of the grooved-anode diode and the simulation method are described in Section 2. The numerical Results and theoretical analysis are given in Section 3. Important conclusions are given in Section 4.

## 2. Device Structure and Simulation Method

**Figure 1:**
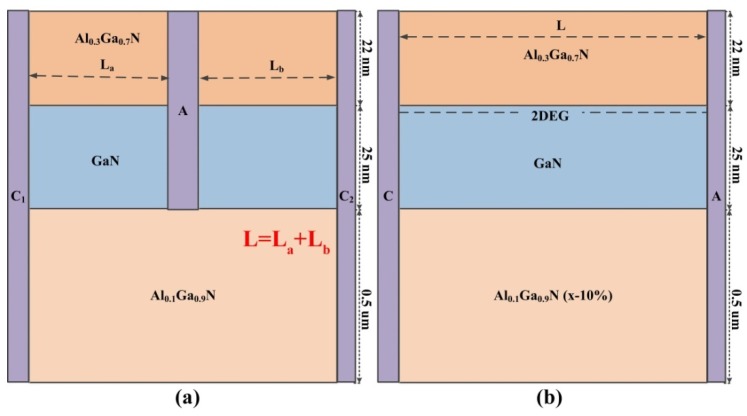
Schematic structures of GaN-based heterostructure Gunn diodes: (**a**) Grooved-anode diode, (**b**) Single-channel diode.

The GaN-based Gunn diode studied in this paper is illustrated in Figure 1. Figure 1a shows the structure of the grooved-anode diode, in which all the structural dimension parameters are labeled clearly. The doping levels of all the material layers are set to be 1 × 10^15^ cm^−3^. The adoption of the Al_0.1_Ga_0.9_N back barrier layer leads to the enhancing confinement of the 2DEG under a high electric field. A rectangular groove is etched through the GaN layer, which ensures that electrons have individual paths in the left and right channels. To achieve the dual-channel diode, a groove-anode area should be defined, and the groove area is etched using a controllable low-damage chlorine-based Inductively Coupled Plasma- Reactive Ion Etching (ICP-RIE) process [25,26]. The definition of the specific technology parameters should be explored further in the actual manufacturing. The anode ohmic contact is deposited in the rectangle grooves and the two cathode ohmic contacts are defined as vertical contacts instead of surface contacts. On one hand, the vertical contacts introduce the lowest parasitic resistance; on the other hand, electric field peaks are easily formed by the surface contact, which results in the premature breakdown of the devices. The total length of the Gunn diode is set to be L. Meanwhile, in the grooved-anode diode, the length of each channel is set to be L_a_ and L_b_ separately, where L_a_ + L_b_ = L. The width of the device is defined as a default of 1 μm in this 2-D simulator. The vertical ohmic contacts can be realized by Molecular beam epitaxy (MBE) regrown technology. In order to simplify the calculation, we performed the simulations under ideal conditions. We assumed that the groove separates the longer channel completely, and the substrate is totally insulated. Therefore, we do not discuss the coupling effect through the substrate here. An energy balance (EB) model with higher order solution of the general Boltzman transport equation was adopted instead of the drift diffusion (DD) model. The energy relaxation time τ_ε_ and momentum relaxation time τ_m_ for GaN 2DEG are defined as 500 and 4 fs, respectively [27,28,29]. The temperature is set to be 300 K, ideally. In order to calculate the RF output characteristics of the Gunn diode, we use the same method as explained in detail in [22,23,24].

In order to calculate the electrical characteristics of the diode, we put a single-tone sinusoidal voltage of form V_DC_ + V_AC_sin(2πft) across the diode instead of embedding it to a resonant circuit, as the external circuit adds complexity to the calculation and easily results in non-convergence. This method is very popular in the analysis of the RF performance of Gunn diodes [30,31,32,33,34,35,36,37] and its validity has been proved in previous publications [33,34,35,36,37]. The applied DC voltage V_DC_ has to be above a critical value so that the device is biased in the negative differential mobility regime. The DC voltage V_DC_ is proportional to length of the diode and V_AC_ = 1/4V_DC_. For example, for the 0.6-µm single channel Gunn diode, V_DC_ = 16 V, V_AC_ = 4 V; for the 1.2-µm single-channel diode, V_DC_ = 32 V, V_AC_ = 8 V; for the 0.6-0.6-µm grooved-anode Gunn diode, V_DC1_ = V_DC2_ = 16 V, V_AC1_ = V_AC2_ = 4 V. The DC-to-AC conversion efficiency η is defined as η = −P_AC_/P_DC_ (P_AC_ is the time-average AC power; P_DC_ is the dissipated DC power).

The AC power delivered is given by [30]

(1) $$P_{\mathrm{AC}}=\frac{V_{\mathrm{AC}}}{T_{R}}\int_{0}^{T_{R}}I\left(t\right)\sin\left(2\pi \mathrm{ft}\right)\mathrm{dt}$$

(2) $$\mathrm{where}\;T_{R}=\frac{1}{f}$$

Similarly, the DC power dissipation in the device is [30]

(3) $$P_{\mathrm{DC}}=\frac{V_{\mathrm{DC}}}{T_{R}}\int_{0}^{T_{R}}i\left(t\right)\mathrm{dt}$$

## 3. Results and Discussion

**Figure 2:**
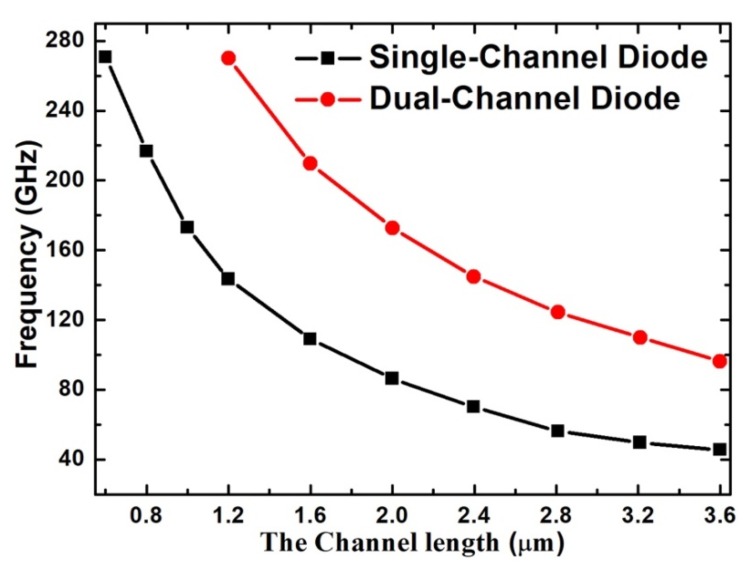
Variation of frequency f with the channel length L in the grooved-anode diode and single-channel diode.

**Figure 3:**
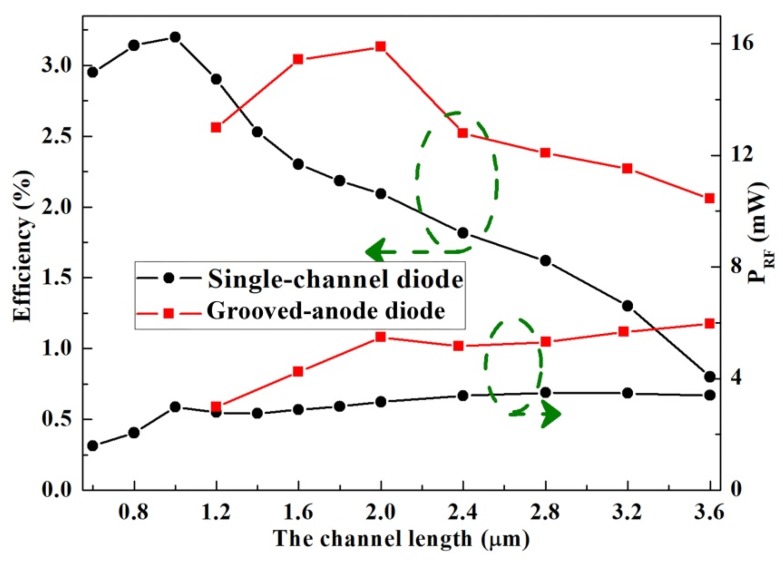
Variation of efficiency η with the channel length L and RF output power P_AC_ with L in the grooved-anode diode and the variation of the single-channel diode.

Both the traditional GaN-based heterostructural Gunn diode with only one channel and the grooved-anode diode were studied as a comparison. In the tradition planar Gunn diode, as the channel length L ranges from 1.2 to 3.6 µm, the frequency f ranges from 143.38 to 45.63 GHz, as shown in Figure 2. The f–L curve of the grooved-anode diode nearly doubles as compared with that of the single-channel diode, as the real channel length of grooved-anode diode is only half of the single-channel diode. The curves also show that f varies inversely to L, which almost matches the formula f = v_sat_/L. In grooved-anode diode, the best output characteristics are achieved at an L of 2.0 µm. The 2.0-µm-grooved-anode diode operates at a frequency f of 172.81 GHz with a DC-to-AC efficiency η of 3.13% and RF output power P_RF_ of 5.48 mW. Meanwhile, the 2.0-µm-single-channel diode generates a frequency of 86.47 GHz with η of 2.09% and P_AC_ of 3.15 mW, as shown in Figure 2 and Figure 3. Therefore, we conclude that the operating frequency and RF output power nearly doubles as compared with the single-channel diode with the same channel length. In addition, the DC-to-AC efficiency η is also greatly enhanced in the grooved-anode diode.

**Figure 4:**
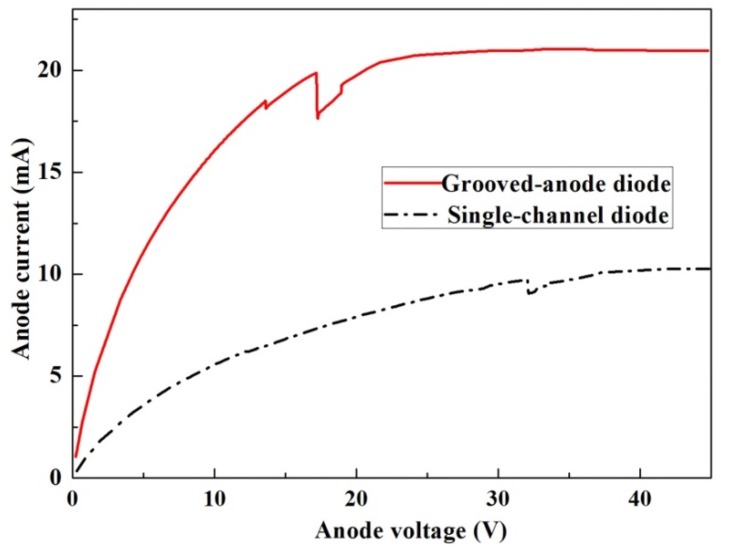
DC I-V output for the grooved-anode diode and single-channel diode as L = 2.0 µm.

**Figure 5:**
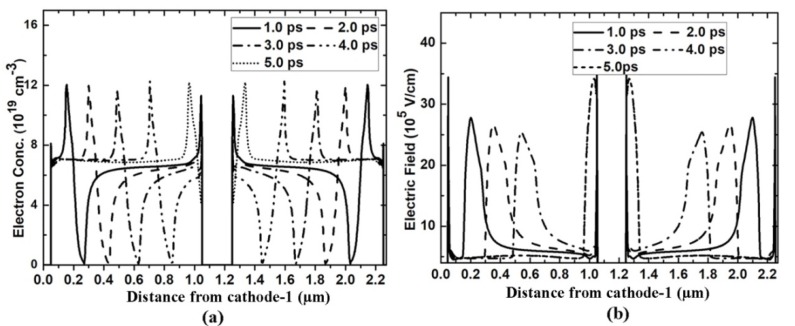
(**a**) Electron concentration and (**b**) Electric field profiles in one oscillation period in the grooved-anode diode as L = 2.0 µm.

Figure 4 gives the I-V characteristics for the grooved-anode diode and single-channel diode of 2.0 µm, from which we can see the grooved-anode diode provides practically the same current, twice the value obtained in the single diode. The grooved-anode diode is equal to two shorter diodes in parallel connection, as the grooved anode is designed in the middle of the diode and divides the long channel into two shorter channels; therefore, the anode current doubles. Perturbation occurs in the I-V characteristics as the applied voltage increases above 17 V in the grooved-anode diode and nearly 34 V in the single-channel diode, which means there are electron domains coming into being in the 2DEG channel. When the anode voltage of the 2.0-µm -grooved-anode diodes increases up to 28 V, prefect stable dipole domains come into being. Figure 5a,b separately give the electron concentration profiles and the corresponding electric field profiles during one oscillating period extracted from the 2.0-µm-grooved-anode diode which show the electron periodic movement during one oscillation period. From Figure 5, we can observe the distinct formation of dipole domains rather than accumulation layers. Theoretically, the dipole domain mode is the most stable mode and generates the highest RF output power and efficiency as compared with other operation modes of the Gunn diode. Based on [13], in order to make the GaN Gunn diode work at a stable dipole domain mode, some conditions should be satisfied [16]:

N_AC_ × L_AC_ > 5 × 10^13^ cm^−2^ (4)

N_AC_ × d > 2 × 10^11^ cm^−2^ (5)

**Figure 6:**
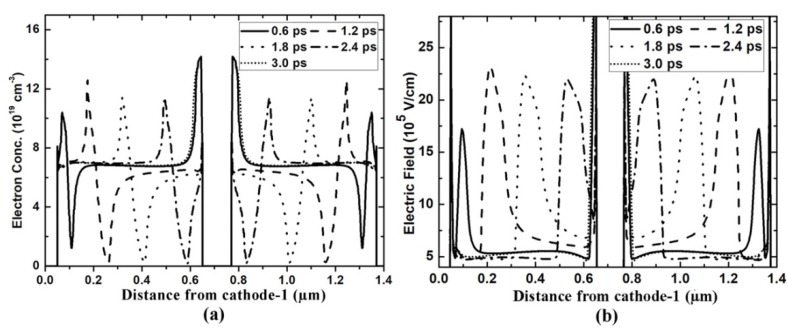
(**a**) Electron concentration and (**b**) Electric field profiles in one oscillation period in the grooved-anode diode as L = 1.2 µm.

N_AC_ is the electron concentration of the active channel, L_AC_ is channel length and d is the channel thickness. These conditions are easily satisfied in GaN-based heterostructure Gunn diode, as in the AlGaN/GaN/AlGaN heterojunction, the electron concentration of 2DEG is up to 10^19^ cm^−3^. Such a high 2DEG is induced by a strong polarization effect of AlGaN/GaN without any doping and well confined in the quantum well. Therefore, 2DEG is well away from the ionized impurity scattering, ensuring the easier formation of the dipole domain in a short channel length. Even when the length of the GaN planar Gunn diode is reduced to several hundred nanometers or the channel thickness is reduced to several nanometers, the diode can still generate stable dipole-domain mode oscillation, as shown in Figure 6. However, a shorter channel length results in a smaller electron domain. In the 1.2-µm-grooved-anode diode, each channel length is 0.6 µm, which is not long enough for the electron dipole to grow mature before exiting from the anode side. Therefore, comparing Figure 5 and Figure 6, especially the Figure 5b and Figure 6b, from the electric field distribution for the 2.0-µm and 1.2-µm diodes we can conclude that a bigger domain forms in the 2.0-µm-grooved-anode diode. Therefore, higher DC-to-AC efficiency is obtained in the 2.0-µm diode.

**Figure 7:**
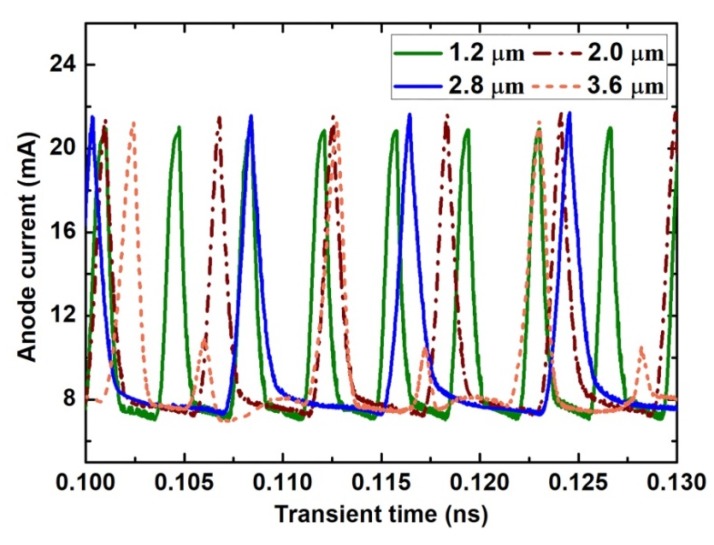
The current oscillation wave in the symmetric grooved-anode diode.

**Figure 8:**
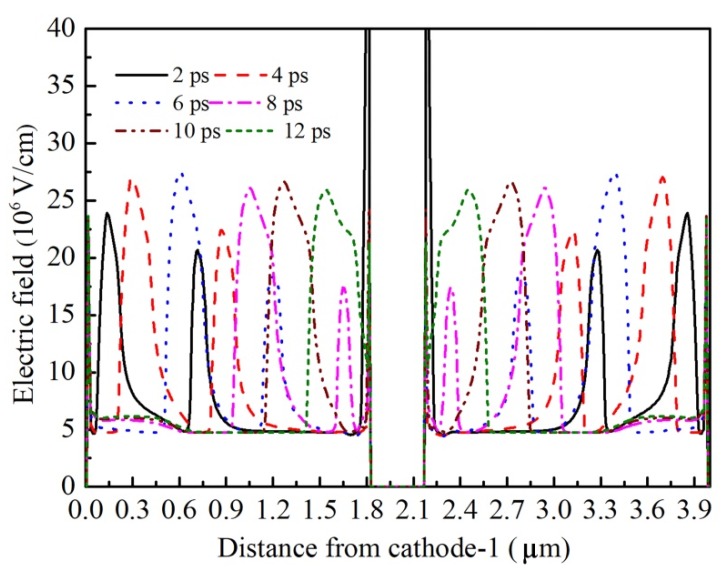
The electric field profiles in one oscillation period in the grooved-anode diode as L = 1.8 µm.

**Figure 9:**
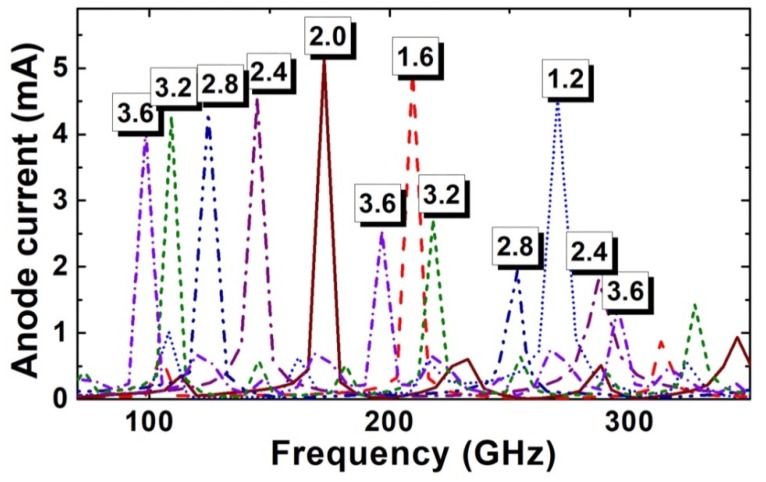
The frequency spectrum diagram of the grooved-anode diode for different L.

Meanwhile, if the channel length is too long, the electron domain grows to its mature size before reaching the anode contact. The remaining channel will be regarded as invalid growth space for the electron domain, which results in the decrease of the efficiency of the Gunn diode. The formation of the electron domain results in the current decline. When the electron domain grows to its full size, the current will drop to its lowest value. If L is too long, the lowest current will last for a period until the electron domain begins to disappear from the anode side. As shown in Figure 7, the lowest-value part of the current wave obviously extends as the channel length L increases, which aggravates the nonlinearity of the oscillation wave, and enhances the harmonic component of the oscillation wave. It is worth noting that two current peaks occur in the 3.6-µm diode, as shown in Figure 7. Figure 8 gives the electron movement tracks in the 3.6-µm diode, which shows that two dipole domains form simultaneously inside each channel; while one forms at near the cathode side, one forms near the middle of the channel. As the electron concentration of 2DEG is very high, therefore, the effective channel lengths for both nucleating points satisfy the condition of the dipole domain. The formation of two domains inside one oscillation circle also aggravates the nonlinearity of the oscillation wave, and weakens the fundamental frequency. In addition, as the two domains restrict each other, neither of them are able to grow to their full size. Therefore, the fundamental and harmonic components of both are reduced. Figure 9 gives the frequency spectrum diagram of the grooved-anode diode for different L from 1.2 to 3.6 µm, which demonstrates that the harmonic component enhances and fundamental component decreases with L. As a result, the noise performance deteriorates with L. In conclusion, in order to avoid the harmonic component enhancement and increase the fundamental component, suitable channel length is of great importance.

**Figure 10:**
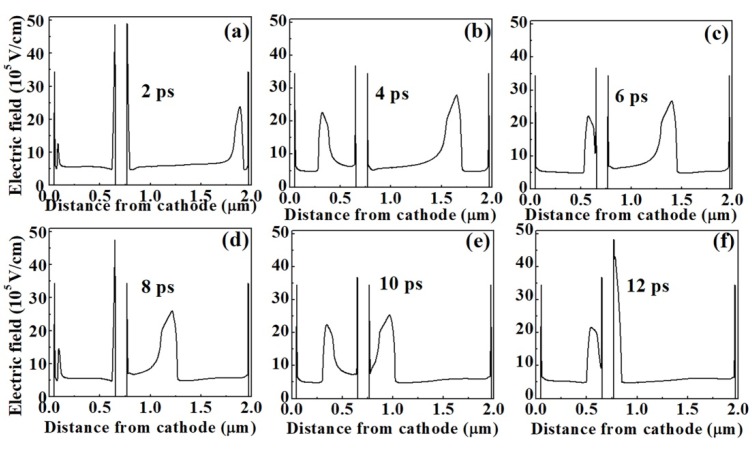
(**a**–**f**) The electric field profiles derived at the same time step in one oscillation period in the grooved-anode diode as L_a_ = 1.0 µm and L_b_ = 2.0 µm.

**Figure 11:**
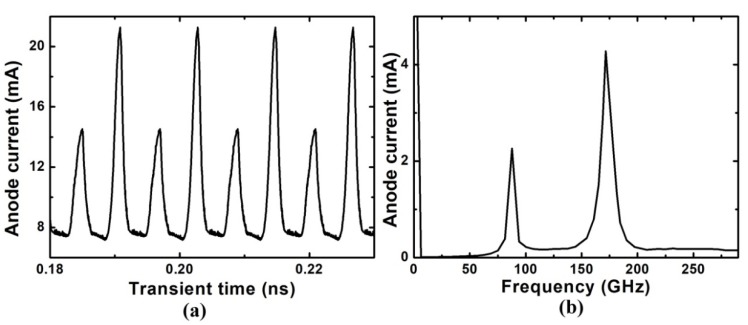
(**a**) The current oscillation wave and (**b**) the frequency spectrum diagram of the grooved-anode diode, where L_a_ = 1.0 µm and L_b_ = 2.0 µm.

We also study the asymmetric grooved-anode Gunn diode, where L_b_ = 2L_a_ = 2.0 µm. In order to ensure that the electric field of each channel is in an appropriate range to generate stable Gunn oscillations, we set V_DC2_ = 2V_DC1_ = 53.4 V, V_AC2_ = 2V_AC1_ = 13.4 V. As the length of the right channel is twice as long as the length of the left channel, the movement period of the electron domain in the right channel should be twice as long as that in the left channel. This is verified by Figure 10, which shows the electric field profiles in one oscillation period derived in the 1.0-2.0-µm-grooved-anode diode. When the dipole domain in the right channel disappears from the anode contact, the dipole domain in the left channel completes two circles, which results in the two current peaks in the current oscillation wave, as shown in Figure 11a. The small current peak is generated as the domain exits from the shorter channel, and the larger current peak is generated as the domain exits from the longer channel. Therefore, two frequencies are obtained, and the second harmonic frequency is greatly enhanced, as shown in Figure 11b. As the two channels are independent of each other, the fundamental and harmonic frequencies are enhanced at the same time. The frequency of the second harmonic is about 175.47 GHz, η is about 1.85%, and P_AC_ is about 4.45 mW. The asymmetric structure realizes the modulation of the operation frequency via a single diode. By changing the length proportion of the two channels, the size and the number of the harmonic wave are able to be controlled.

## 4. Conclusions

In this paper, for the first time we propose the grooved-anode planar Gunn diode to greatly enhance the RF output power at a high operation frequency. We present an explicit numerical study into its working principle and output characteristics based on a simulation method. The grooved-anode diode is equal to two shorter diodes in parallel connection, as the grooved anode divides the long channel into two shorter channels. In the symmetric grooved-anode diode, the RF output power is almost doubled in the grooved-anode diode as compared with the single-channel diode. The 1.0-1.0-µm grooved-anode diode shows the best output characteristics. It operates at a fundamental frequency of 172.81 GHz and the corresponding DC-to-AC conversion efficiency is about 3.13%. It produces over 5.48 mW of power, nearly twice as high as that of the 1.0-µm single-channel diode. This novel grooved-anode diode realizes the enhancement of the frequency and RF output power simultaneously, by simply etching a rectangular grooved anode onto the semiconductor layer at one lithographic step, which provides good design ideas in improving the output characteristic of the terahertz sources and other power devices. In the asymmetric 1.0-2.0-µm grooved-anode diode, two frequencies are obtained, and the second harmonic is enhanced as compared with the fundamental wave. The harmonic-enhanced Gunn diode shows its potentials as a mixer or frequency multiplier. Furthermore, it will provide a fast conversion between two different frequencies without connecting with other terahertz oscillators. We have demonstrated that the proposed GaN heterostructure grooved-anode planar Gunn diode is an excellent candidate as a solid-state terahertz device.
